# Surface Area Graphic Evaluation (SAGE) Diagram Documentation in Burn Patients: Room for Quality Improvement

**DOI:** 10.7759/cureus.13731

**Published:** 2021-03-06

**Authors:** Mattalynn Chavez-Navin, Barkat Ali, EunHo Eunice Choi, Ryan Keffer, Sydney Cooper, Whitney Elks, Victor Andujo, Gregory Borah

**Affiliations:** 1 General Surgery, The University of New Mexico Health Sciences Center, Albuquerque, USA; 2 Surgery, The University of New Mexico Health Sciences Center, Albuquerque, USA; 3 Biostatistics, The University of New Mexico Health Sciences Center, Albuquerque, USA; 4 General Surgery, Baylor Scott & White Health, Temple, USA; 5 General Surgery, University of Nevada, Las Vegas (UNLV), Las Vegas, USA; 6 General Surgery, Oregon Health and Science University School of Medicine, Portland, USA; 7 Surgery, University of New Mexico School of Medicine, Albuquerque, USA

**Keywords:** sage diagram documentation, burn, quality improvement

## Abstract

Background

The first step in the management of burn patients is an accurate estimation of the total body surface area (TBSA) involvement. Depending on which, burns are categorized as major (>20%) and minor (<20%). This then dictates fluid resuscitation and level of care. At the University of New Mexico Burn Center, we use Surface Area Graphic Evaluation (SAGE) diagramming to objectively estimate the body surface area involvement. We hypothesized patients undergoing SAGE documentation will have better outcomes.

Methods

This is a retrospective study of 320 consecutive patients from 2014-2018 at the University of New Mexico Burn Center. Only patients treated surgically were included. We recorded patient demographics, comorbidities, and burn details. The primary measure of interest was SAGE documentation and the secondary measure of interest was outcomes associated with it.

Results

We found that a SAGE diagram was only documented for a minority of patients (40%). After comparing patients in the SAGE group vs. No SAGE group, we found that the patients were the same in both groups with regards to demographics, comorbidities, and burn characteristics. The use of a SAGE diagram did not appear to be a significant predictor of complications, including surgical site infections, graft loss, donor site complications, postoperative pneumonia, urinary tract infections, deep vein thrombosis, or myocardial infarction (p=0.254).

Conclusion

Only a minority of patients get a SAGE diagram documented. However, our study did not find any improved outcomes with the use of a SAGE diagram. There is a need for prospective studies to validate the utility of SAGE diagramming in predicting adverse outcomes in major burns.

## Introduction

The first step in the management of acute burn patients is an accurate estimation of the total body surface area (TBSA) involvement [1]. Based on the TBSA i.e. % body surface area, burns are categorized as major (>20% TBSA) and minor (<20% TBSA) [2]. The calculated TBSA dictates further management. An accurate estimation of TBSA is crucial in the immediate evaluation of patients since the patients with major burns require fluid resuscitation in the monitored unit [3].

The total body surface involvement can be subjectively or objectively estimated. The subjective means of estimation is clinical assessment while the object means is through the use of computerized diagramming tools such as the Surface Area Graphic Evaluation (SAGE) Diagram, LLC [4]. The SAGE Diagram is a free online program designed to be a convenient and accurate tool to calculate burn extent, depth, and %TBSA [5]. Following an accurate determination of TBSA involvement, resuscitation protocol ensues based on the Parkland equation for major burns [6].

Despite knowing that the inaccurate prediction of burn surface area involvement can delay resuscitation measures and potentially lead to increased mortality and morbidity [7], there is much variability in the method to calculate burn extent between hospitals and providers.

To our knowledge, the clinical efficacy of the online SAGE diagram tool has not been previously been demonstrated. The aim of this study was to investigate the functional outcomes associated with the utility of the SAGE diagram by comparing %TBSA estimates between clinical estimates and that using the SAGE diagram.

## Materials and methods

We performed a retrospective chart review of burn patients treated surgically at a designated burn center between 2014 and 2018. The study group included all individuals who received immediate debridement and homograft placement and patients who underwent staged excision before autograft placement. Exclusion criteria included patients who were treated non operatively. Data collection comprised of demographics, medical comorbidities, burn mechanism, burn degree, and depth of burn injury. The primary variable of interest was SAGE documentation as recorded in the electronic medical records. The patients were divided into two groups based on SAGE documentation. Group comparisons were performed using descriptive statistics. The level of significance was set at p < 0.05.

This documentation includes the patient’s name, medical record number, age, and weight and height in centimeters. After creating a profile, the user will be shown a front and back prototype diagram of the patient. The operator can manually draw in the area of burn correlating to the body part(s) involved. After shading the appropriate body part involvement, the program will calculate %TBSA based on the Lund-Browder method. The image can then be printed and incorporated into the patient’s chart as part of critical documentation.

## Results

Of the 320 total patients, SAGE was documented for only a minority of our patient population (40%). After comparing patients in SAGE group vs. No SAGE group, we found that the patients were the same in both groups with regards to the demographics, comorbidities, and burn characteristics. Table 1 describes our patient population demographics. There was no significant difference between the SAGE group versus the No SAGE group when comparing age distribution (p=0.478). The majority of our patient population were adults, age >18, and they were predominately male (>70%). 30% of patients were obese in both groups. There was no difference regarding race or skin color between the two groups (p=0.130). There was also no statistically significant difference between admission status (intensive care unit vs floor) that determined whether or not the patient received a SAGE diagram (p=0.248). There was no significant difference between the two groups regarding disposition home versus to skilled-nursing facilities, long-term care centers, or death (p=0.204). However, we did find a statistically significant difference between those who had SAGES versus No SAGES in regard to median total operating room (OR) time (p <0.001).

**Table 1:** Demographics *Indicates some data was not available in documentation. SAGE: Surface Area Graphic Evaluation; BMI: body mass index; COPD: chronic obstructive pulmonary disease; ICU: intensive care unit.

Variable	Total patients	No SAGE	SAGE	P-value
	n=320	n=192	n=128	
Age group				
Pediatrics	47	26	21	0.478
Adults	273	166	107	
Gender				
Male	234	142	92	0.68
Female	86	50	36	
Obesity				
BMI<30	199	119	80	0.995*
BMI>30	82	49	33	
Race				
White/Hispanic	208	129	79	0.13
Minority	75	39	36	
Comorbidities				
Diabetes	62	37	25	0.954
Hypertension	77	45	32	0.749
Hyperlipidemia	36	21	15	0.828
Coronary artery disease	15	10	5	0.788
Peripheral Vascular Disease	5	1	4	0.085
Renal Failure	5	2	3	0.393
Chronic Liver Disease	18	10	8	0.692
IV drug use	14	8	6	0.823
Smokers	94	62	32	0.164
COPD	19	11	8	0.847
Admission Level				
Floor	239	139	100	0.248
ICU	81	53	28	
Disposition				
Home	230	143	87	0.204
Other facility	90	49	41	

We next looked at the burn characteristics between the two groups described in Table 2. Burn mechanisms that were noted ranged from scald, thermal, flash, explosion, road rash, friction, de-gloving, chemical, and electrical burns. There was no statistically significant difference between the two groups when comparing burn mechanism (p=0.665). Burns were categorized as major (TBSA >20%) and minor (TBSA <20%). We found that there was no statistical difference between SAGE versus No SAGE groups regarding extent of burn surface area involvement (p=0.613). We also wanted to determine if the presence of burn cellulitis at admission would determine whether or not a patient received a SAGE diagram. We found no difference between the two groups with respects to burn cellulitis at admission (p=0.142).

**Table 2:** Burn characteristics SAGE: Surface Area Graphic Evaluation; TBSA: total body surface area.

Variable	Total	No SAGE n=192	SAGE n= 128	P value
Burn Mechanism				0.665
Scald	81	42	39	
Thermal	140	87	53	
Flash/explosion	52	32	20	
Road Rash/Friction/De-gloving	24	14	10	
IV infiltration/Chem/Electrical	23	17	6	
TBSA				
Minor (<20)	284	169	115	0.613
Major (>20)	36	23	13	
Burn cellulitis at admission	117	64	53	0.142
Involvement by body parts				
Face	54	34	20	0.626
Scalp	2	2	0	0.516
Neck	25	14	11	0.671
Anterior Torso	87	57	30	0.218
Upper Extremity	152	91	61	0.964
Hands	125	82	43	0.102
Posterior Torso	41	30	11	0.065
Lower Extremity	204	116	88	0.129
Buttock	15	11	4	0.419
Perineum	9	6	3	0.746
More than 1 body part involved	107	63	44	0.772
Graft take				
<100%	138	91	47	0.059

Next, we wanted to determine if certain body part involvements influenced SAGE diagramming or not. There was no significant difference between those who received SAGE vs No SAGE with regards to body part involvement (face, p=0.626, scalp, p=0.516, neck, p=0.671, anterior torso, p=0.218, upper extremity, p=0.964, hands, p=0.102, posterior torso, p=0.065, lower extremity, p=0.129., buttock, p=0.419, perineum, p=0.746). There was no significant difference in the use of diagramming when more than one body part was found to be involved (p=0.772). 

Percent graft take after surgery was compared between patients who received SAGE diagram to those who did not. Graft take percentage was determined by an experienced burn surgeon and was categorized as either 100% take versus less than 100% take. The percent graft take was not significantly different between the two groups (p=0.059).

Complication development was compared between the two groups, as seen in Table 3. Complications included post-operative fevers, urinary tract infections, pneumonia, thrombotic evens, surgical site infections, skin graft loss, or issues at the donor site. We found no difference in complications between those who received SAGE diagramming versus those who did not (p=0.254).

**Table 3:** Complications between SAGE vs No SAGE SAGE: Surface Area Graphic Evaluation.

	Total	No SAGE n=192	SAGE n=128	p-value
Any complication				
Yes	49	33	16	0.254
No	271	159	112	

**Figure 1:**
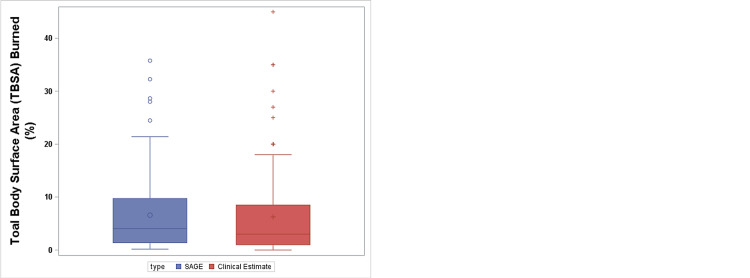
SAGE diagram vs clinical estimate %TBSA Using the Wilcoxon rank test, there is no statistically significant difference between the %TBSA determined via clinical estimate to that determined by the use of SAGE diagram. p=0.076 SAGE: Surface Area Graphic Evaluation; TBSA: total body surface area.

Finally, we compared the clinical estimate of %TBSA to that determined by the use of SAGE diagram. Using the Wilcoxon rank test, we found no statistically significant difference between the %TBSA determined clinically followed by the use of a SAGE diagram demonstrated in Figure 1 (p= 0.076).

## Discussion

Burn injuries account for 180,000 deaths per year and are a leading cause of morbidity [8]. While the mechanisms of burn injuries can vary, the first step in managing acute burns is accurate estimation of the patient’s TBSA involved. This is imperative for fluid resuscitation, initiating referrals to burn centers, and can be detrimental in morbidity and mortality [9]. Inappropriate estimation of burn extent can have adverse outcomes for patients such as administration of inadequate fluid resuscitation or lead to costly transfers that could have otherwise been avoided [10]. Despite understanding the complexity of burn care and our knowledge regarding the importance of initial %TBSA calculation, better burn estimation modalities and policies are still not universally adopted.

Burns are classified as major (>20% TBSA) or minor (<20% TBSA). The evaluation of burns can be subjective via clinical assessment or objective through body mapping tools. The most widely used methods to estimate burn extent include The Rule of Nines, Rule of Palm, and the Lund-Browder chart. The Rule of Nines provides a quick way to estimate medium to large-sized burns and functions on the principle that the body is divided into areas of 9%. This method has limited utility in the pediatric population [11]. The Rule of Palm method predicts %TBSA by assuming that the surface area of a patient’s palm is about 0.8% of their TBSA and is best used in the estimation of smaller burns [11] Of the three methods, the Lund-Browder chart is the most accurate and can be used for both adults and children. The Lund-Browder chart provides both an anterior and posterior diagram of the human body with sections of the body labelled with their respective percent body surface area. The evaluator can shade in the affected area and use the illustration to help estimate the %TBSA involved. It is important to note that simple erythematous areas should not be included in the calculation, which can be a common error made by evaluators [12]. 

Computer diagraming tools have been developed to assist clinicians in the evaluation of burns to mitigate subjective errors in estimation. For example, the SAGE Diagram, LLC described earlier is a two-dimensional computer program that utilizes the Lund-Browder principle to calculate a %TBSA and can be useful when more than one body part is involved. Although computer programs are arguably more accurate than other subjective means of %TBSA estimation, there are still limits to utilizing these tools especially with regards to overweight patients and infants [13]. Even with access to various computer programs, several studies have shown that clinicians are still poor predictors of %TBSA and burn depth. Studies show that even experienced surgeons have between a 64%-76% success rate in accurate determination of burn depths [11,14]. In the emergency department, overestimation of burn injuries has been reported to occur by a factor greater than 100% [13].

In our retrospective study, we showed that a vast majority of our burn population did not receive SAGE diagramming despite being mandated for a designated burn center. There could be several potential reasons for this nonadherence. Firstly, it can be a cumbersome task and requires extra time to perform if doing correctly. Secondly, lack of logistics e.g. not every computer has the appropriate software downloaded to run the program, difficulty uploading the diagram onto the patient’s medical chart after printing. This can also be a limitation to our study since clinicians may have utilized SAGE diagramming but did not document this appropriately. Finally, our results suggest that SAGE diagramming provided no additional benefit in %TBSA estimation given no statistically significant difference in estimation between clinical estimate and that produced by this online program.

There is potential to further investigate the functional utility of SAGE diagramming. However, given the vast systems issues associated with SAGE diagramming and its limited 2D features, this tool is outdated and inefficient. Newer technology has been developed to better predict TBSA involvement compared to SAGE diagramming. Three-dimensional %TBSA mapping programs have previously been explored and demonstrate enhanced prediction of burn extent in children, obese, and female patients with favorable usability [15]. 3D wound mapping technology may be an even more useful tool in settings comprised mainly of trainees since this tool can be handheld and %TBSA estimations are shown to be agreeable to that of experts [16].

## Conclusions

In order to be a designated burn center, all burn patients are required to have documentation of %TBSA involvement. The online SAGE diagram program is one example of a tool that allows practitioners to estimate %TBSA. However, our retrospective chart review demonstrated that the vast majority of burn patients did not have SAGE diagramming completed or documented. We found no significant difference between the SAGE diagram group versus the No SAGE group regarding functional outcomes and clinically estimated %TBSA, rendering this tool to be inefficient. Therefore, there is a need for a more robust, standardized, and accessible tool to better predict %TBSA in burn populations given that the %TBSA value is critical in the management of burn patients and their potential transfer to an outside facility.
